# Pneumococcal conjugate vaccination schedules in infants—acquisition, immunogenicity, and pneumococcal conjugate and yellow fever vaccine co-administration study

**DOI:** 10.1186/s13063-021-05949-4

**Published:** 2022-01-15

**Authors:** Grant A. Mackenzie, Isaac Osei, Rasheed Salaudeen, Ousman Secka, Umberto D’Alessandro, Ed Clarke, Jonas Schmidt-Chanasit, Paul V. Licciardi, Cattram Nguyen, Brian Greenwood, Kim Mulholland

**Affiliations:** 1 Medical Research Council Unit The Gambia at London School of Hygiene & Tropical Medicine, Fajara, The Gambia; 2 Faculty of Infectious & Tropical Diseases, London School of Hygiene & Tropical Medicine, London, UK; 3 Murdoch Children’s Research Institute, Melbourne, Australia; 4 Department of Paediatrics, University of Melbourne, Melbourne, Australia; 5 Bernard Nocht Institute for Tropical Medicine, Hamburg, Germany; 6 Faculty of Epidemiology and Public Health, London School of Hygiene & Tropical Medicine, London, UK

**Keywords:** Cluster-randomised controlled trial, Pneumococcal, Vaccine, Schedule, Immunogenicity, Acquisition

## Abstract

**Background:**

Pneumococcal conjugate vaccines (PCVs) effectively prevent pneumococcal disease, but the global impact of pneumococcal vaccination is hampered by its cost. The evaluation of reduced dose schedules of PCV includes measurement of effects on immunogenicity and carriage acquisition compared to standard schedules. The relevance and feasibility of trials of reduced dose schedules is greatest in middle- and low-income countries, such as The Gambia, where the introduction of PCV resulted in good disease control but where transmission of vaccine-type pneumococci persists. We designed a large cluster-randomised field trial of an alternative reduced dose schedule of PCV compared to the standard schedule, the PVS trial. We will also conduct a sub-study to evaluate the individual-level effect of the two schedules on carriage acquisition, immunogenicity, and co-administration of PCV with yellow fever vaccine, the PVS-AcqImm trial.

**Methods:**

PVS-AcqImm is a prospective, cluster-randomised trial of one dose of PCV scheduled at age 6 weeks with a booster dose at age 9 months (i.e. alternative ‘1+1’ schedule) compared to three primary doses scheduled at 6, 10, and 14 weeks of age (i.e. standard ‘3+0’ schedule). Sub-groups within the alternative schedule group will receive yellow fever vaccine separately or co-administered with PCV at 9 months of age. The primary endpoints are (a) rate of nasopharyngeal vaccine-type pneumococcal acquisition from 9 to 14 months of age, (b) geometric mean concentration of vaccine-type pneumococcal IgG at 18 months of age, and (c) proportions with yellow fever neutralising antibody titre ≥8 four weeks after administration of yellow fever vaccine. Participants and field staff will not be masked to group allocation while the measurement of laboratory endpoints will be masked. Approximately equal numbers of participants will be resident in each of 28 geographic clusters (14 clusters in alternative and standard schedule groups); 784 enrolled for acquisition measurements and 336 for immunogenicity measurements.

**Discussion:**

Analysis will account for potential non-independence of measurements by cluster and so interpretation of effects will be at the individual level (i.e. a population of individuals). PVS-AcqImm will evaluate whether acquisition of vaccine-type pneumococci is reduced by the alternative compared to the standard schedule, which is required if the alternative schedule is to be effective. Likewise, evidence of superior immune response at 18 months of age and safety of PCV co-administration with yellow fever vaccine will support decision-making regarding the use of the alternative 1+1 schedule. Acquisition and immunogenicity outcomes will be essential for the interpretation of the results of the large field trial comparing the two schedules.

**Trial registration:**

International Standard Randomised Controlled Trial Number 72821613.

**Supplementary Information:**

The online version contains supplementary material available at 10.1186/s13063-021-05949-4.

## Administrative information

Note: the numbers in curly brackets in this protocol refer to SPIRIT checklist item numbers [1]. The order of the items has been modified to group similar items (see http://www.equator-network.org/reporting-guidelines/spirit-2013-statement-defining-standard-protocol-items-for-clinical-trials/) (Table 1).

**Table 1:** Administrative information showing key SPIRIT checklist items

**Title {1}**	Pneumococcal conjugate vaccination schedules in infants - acquisition, immunogenicity and pneumococcal conjugate and yellow fever vaccine co-administration study
**Trial registration** **{2a} and {2b}**	International Standard Randomised Controlled Trials Number – 72821613, 10.1186/ISRCTN72821613. Registered on 28 November 2019.
**Protocol version {3}**	Protocol version 5.0, 24 May 2021
**Funding {4}**	Bill & Melinda Gates Foundation (INV006724); Mucosal Pathogens Research Unit, University College London (Ref 5356358), National Institute of Health Research (UK); Medical Research Council Unit The Gambia at London School of Hygiene & Tropical Medicine.
**Author details {5a}**	Grant A Mackenzie^1,2,3,4^, Isaac Osei^1,2^, Rasheed Salaudeen^1^, Ousman Secka^1^, Umberto D’Alessandro^1^, Ed Clarke^1^, Jonas Schmidt-Chanasit^5^, Paul V Licciardi^3^, Cattram Nguyen^3^, Brian Greenwood^2^, Kim Mulholland^3,4,6^ 1 Medical Research Council Unit The Gambia at London School of Hygiene & Tropical Medicine, Fajara, The Gambia. 2 Faculty of Infectious & Tropical Diseases, London School of Hygiene & Tropical Medicine, London, UK. 3 Murdoch Children’s Research Institute, Melbourne, Australia. 4 Department of Paediatrics, University of Melbourne, Australia. 5 Bernard Nocht Institute for Tropical Medicine, Hamburg, Germany 6 Faculty of Epidemiology and Public Health, London School of Hygiene & Tropical Medicine, London, UK.
**Contact information for trial sponsor {5b}**	London School of Hygiene & Tropical Medicine, Keppel Street, London, WC1E 7HT, UK. Contact name: Head of Research Governance and Integrity, RGIO@lshtm.ac.uk.
**Role of sponsor and funder {5c}**	The trial sponsor and funders are not involved in the study design; collection, management, analysis and interpretation of data; writing of the report; the decision to submit the report for publication, and will not have authority over any of these activities.

## Introduction

### Background and rationale {6a}

Despite the pneumococcus causing more childhood deaths than any single pathogen [2, 3], global control of pneumococcal disease is hampered by the cost of pneumococcal conjugate vaccines (PCVs). In addition to the relatively high cost of several new vaccines that have recently been introduced in many low-income countries, expanded programmes on immunisation (EPI) face the additional challenge of schedules with increasing numbers of doses. Reducing the cost and complexity of EPI schedules would improve the flexibility, acceptability, and sustainability of immunisation programmes.

Low-income countries receive subsidised PCV through the GAVI Alliance, providing a co-payment of 0.15–0.30 USD per dose (increasing 15% per year in ‘intermediate’ countries) [4]. However, when countries’ Gross National Income per capita exceeds the World Bank ‘low-income’ threshold of ~ 1500 USD, they begin to transition from GAVI support. During transition, co-payments increase each year for 5 years to a final price set under the GAVI Advance Market Commitment (2.00–2.90 USD per dose) [5]. GAVI expenditure on PCV represents approximately half of its vaccine budget [6]. The importance of the cost of PCV was evident in The Gambia where its introduction, at 0.2 USD per dose, increased the national cost of the EPI programme by one-third, with vaccine representing 91% of the total cost of introducing PCV [7]. Thus, a major determinant of the sustainability of pneumococcal vaccination in low- and middle-income countries is cost. Middle-income countries experience many child deaths due to pneumococcus but cost has precluded many from introducing PCV.

EPI programmes in low- and middle-income countries are becoming more complicated and costly with the introduction of new vaccines. The addition of vaccines such as PCV, rotavirus vaccine, injectable polio vaccine, meningococcal group A conjugate, human papillomavirus vaccine, and typhoid conjugate challenges the implementation, acceptance, cold-chain capacity, and sustainability of EPIs. The difficulty of introducing such new vaccines has its biggest impact in low- and middle-income countries where the burden of disease is greatest but resources are scarce.

The Medical Research Council Unit The Gambia at London School of Hygiene & Tropical Medicine (MRCG at LSHTM) has a long history investigating the burden of pneumococcal disease and pneumococcal vaccination. In 2000–2004, a trial of a 9-valent PCV (PCV9) was conducted in Central and Upper River Regions (CRR and URR) of The Gambia. Vaccine efficacy in children aged 3–29 months was 37% against radiological pneumonia, 77% against vaccine-type (VT) invasive pneumococcal disease (IPD), and 16% against all-cause mortality [8]. In 2009, The Gambia introduced PCV7 into the routine EPI using a three-dose schedule without a booster dose (i.e. a ‘3+0’ schedule). In 2011, PCV7 was replaced by PCV13. The Pneumococcal Surveillance Project (PSP) has documented the impact of PCV13 in the Basse Health & Demographic Surveillance System (BHDSS) in rural Gambia. Four to 5 years after the introduction of PCV7/13, the incidence of VT IPD had declined by 82%, with a 24% reduction in radiological pneumonia and 61% reduction in severe hypoxic pneumonia in children aged 2–59 months [9, 10]. Eight years after the introduction of PCV7/13, the incidence of VT IPD in the 2–59-month age group has declined by 92% and radiological pneumonia has declined by 27% [11]. Before the introduction of PCV, PSP detected an average of 35 annual cases of VT IPD among children aged 2–59 months. In 2016, we detected six cases of VT IPD, and in 2017, we detected three. In 2016/2017, we detected zero cases of VT IPD among children in the first year of life. These data indicate that the introduction of PCV7/13 has now controlled VT IPD.

It is now evident that following the introduction of PCV13, herd protection has developed in The Gambia. The annual count of VT IPD in older children in PSP was six to ten before the introduction of PCV13 in 2011. Following the introduction of PCV13, the annual case counts in 2015, 2016, and 2017 were four, one, and zero, respectively (author’s own data). In the 5–14-year age group, IPD incidence declined by 69% (95% *CI*, −28–91%) and radiological pneumonia by 27% (95% *CI*, −5–49%) [11].

The prevalence of nasopharyngeal (NP) carriage of PCV13 VT in the BHDSS area before the introduction of vaccine was 47% in the under-5-year age group. In 2015 and 2017, the prevalence of vaccine types was 17% and 15%, respectively (author’s own data). The downward trajectory of vaccine-type prevalence from 2009 to 2015 and 2017 suggests that the introduction of PCV13 has substantially reduced the prevalence of VT carriage. However, it is evident that transmission of VT pneumococci continues in the population.

The Gambian EPI schedule currently includes birth doses of BCG, hepatitis B, and oral polio vaccine (OPV); visits at 2 and 3 months of age when OPV, pentavalent, rotavirus, and PCV13 vaccines are scheduled; a visit at 4 months of age when OPV, injectable polio (IPV), pentavalent, rotavirus, and PCV13 vaccines are scheduled; a visit at 9 months of age when measles-rubella and yellow fever vaccines are scheduled; conjugate meningococcal group A vaccine scheduled at 12 months of age was introduced in 2018; at 18 months of age, OPV and measles-rubella vaccines are scheduled. Human papilloma virus vaccine delivered to school-age girls was introduced in 2020. In the event that polio is eradicated, then OPV will be phased out and be replaced by IPV. The EPI is also considering the introduction of a conjugate typhoid vaccine. Thus, in recent years, the EPI schedule has introduced several additional injectable antigens and more may be added in the future.

Several studies indicate that immunological priming for an optimal PCV booster dose-response may be more effective with fewer primary doses [12, 13]. In addition, the immunological response to a booster dose following a single priming dose may reduce VT acquisition to a greater degree than following the standard 3+0 schedule [14]. As a result, a schedule with one primary dose and a later booster dose, that is a 1+1 schedule, may induce greater herd protection than the 3+0 schedule. A potential barrier to the use of booster doses of PCV in low-income countries is the lack of evidence of safe co-administration with the YF vaccine, which in Africa is generally scheduled at 9 months of age.

This trial joins a global initiative to generate evidence about reduced dose schedules for PCV. WHO is engaged with this initiative having held a consultative meeting in February 2016. Studies investigating reduced dose schedules for PCV are underway in South Africa, Vietnam, India, and the UK. The UK introduced a 1+1 schedule nationwide in 2019. Our trial in The Gambia is critical to provide evidence from a typical African setting.

In Fiji and The Gambia, one dose of PCV at 2 or 3 months of age significantly reduced carriage of VT pneumococci at 9 months of age [13, 15]. A Dutch study showed that following two doses of PCV7 at the ages of 2 and 4 months, a booster dose at 11 months prevented VT carriage in the 2nd year of life [14].

Data from a recent UK trial show that the immunogenicity of the PCV booster dose using a 1+1 schedule was equivalent to, or superior to, a 2+1 schedule for nine of the 13 serotypes in PCV13 [12]. Of importance to the Gambian setting, where serotypes 1 and 14 have been the most common serotypes causing IPD, IgG responses to those serotypes following the booster dose were superior in the 1+1 group. Almost all infants in both the 1+1 and 2+1 groups had IgG responses above the protective titre of 0.35 μl/ml, for all serotypes except serotype 3, for which fewer reached protective thresholds in both schedules. Geometric mean IgG concentrations following the primary series were higher in the 2+1 schedule, although differing durations of time between vaccination and blood collection biased the results towards lower concentrations in the 1+1 group. The UK transitioned to a national 1+1 schedule in 2019 [16]. There is also suggestive clinical evidence that herd protection following the use of a 2+1 schedule is greater than with a 3+0 schedule [17].

The duration of protection of PCV is poorly defined, but the potential for greater antibody persistence following a booster dose compared to doses in early infancy [18, 19] suggests that protection may be more long-lived when a booster dose is given [20]. Even though PCV has proven efficacious against serotype 1 in young African children, serotype 1 continues to cause epidemic meningitis in the African meningitis belt [21]. There is a strong rationale to test, in our current epidemiological setting, whether a 1+1 schedule will provide an overall non-inferior programmatic effect compared to the 3+0 schedule. It is important to note that as the time course after the introduction of a vaccine extends, the direct effect of vaccination becomes less important and herd protection assumes an increasingly important role [22]. This trial aims to conduct an immunogenicity and acquisition sub-study within a larger pneumococcal vaccine schedule (PVS) field trial. Immunogenicity data will be important to interpret the results of the field trial. Immunogenicity data on the 1+1 schedule are needed in a typical African population given that administration of the first dose is likely to be earlier than in developed countries, concentrations of maternally derived antibody, which may affect responses to the primary dose(s), are different in populations with high pneumococcal transmission, and responses to PCV may be quantitatively different in these compared with other populations. Similarly, empiric measurement of the effect of the PCV13 booster dose on VT acquisition will provide direct evidence of the relative effect of the two schedules on herd protection and assist interpretation of the main PVS trial.

Demonstrating a superior immune response at 18 months of age and reduced VT acquisition following the booster dose will provide supporting evidence to the PVS field trial assisting decision-makers in considerations of reduced dose schedules. Reducing the number of PCV doses in EPI schedules will impact on multiple elements of the challenges posed by this vaccine: reducing the costs to countries and GAVI, reducing the number of injections in schedules and providing greater flexibility for the inclusion of other vaccines, reducing staff time and cold-chain requirements, and ultimately making EPI programmes more acceptable and sustainable. If countries can safely transition to 1+1 schedules, the global uptake of PCV should accelerate with greater and more sustainable reductions in pneumococcal disease.

### Objectives {7}

The primary objectives will be achieved by testing the following hypotheses:

• The serotype-specific IgG concentration for VT at 18 months of age will be greater in 1+1 compared to 3+0 schedule recipients.

• The rate of NP VT acquisition from 9 to 14 months of age will be reduced in 1+1 compared to 3+0 schedule recipients.

• The proportion of participants with protective titres of yellow fever (YF) neutralising antibodies will be non-inferior in those who receive co-administered compared to separately administered PCV and yellow fever vaccines.

The secondary objectives are to compare in alternative versus standard schedule groups the:

• Rate of NP acquisition of non-VT pneumococci between 9 and 14 months of age

• Proportion of participants with NP VT colonisation at 6, 9, and 18 months of age

• Proportion of participants with pneumococcal VT IgG concentration ≥0.35 μg/ml, 4 weeks after the primary series, 4 weeks after the booster dose at age 9 months, and at 18 months of age

• Pneumococcal VT opsonophagocytic antibody (OPA) titres following a single dose at age 6 weeks, following three primary doses, following the booster dose at age 9 months, and at 18 months of age

• Geometric mean concentrations (GMCs) of pneumococcal VT IgGs 4 weeks after administration of PCV13 at 9 months of age with and without co-administration with the YF vaccine

### Trial design {8}

The PVS acquisition/immunogenicity (PVS-AcqImm) is a parallel group, unmasked, cluster-randomised trial of the individual-level effect (i.e. a population of individuals) of two different schedules of PCV13. This trial is nested within the PVS field trial but designed for interpretation of effects at the individual level. This is a phase IV trial involving licenced products comparing alternative and standard schedules for PCV13 and separate versus co-administration with the YF vaccine. We will test the superiority of the 1+1 compared to the 3+0 schedule to reduce the rate of NP acquisition of VT pneumococci from 9 to 14 months of age and in terms of the immune response at 18 months of age. We will test the non-inferiority of the immune response following separate and co-administration of PCV and YF vaccines. Approximately equal numbers of participants will be enrolled in each cluster allocated to the two groups in a 1:1 ratio.

### Study setting {9}

**Fig. 1:**
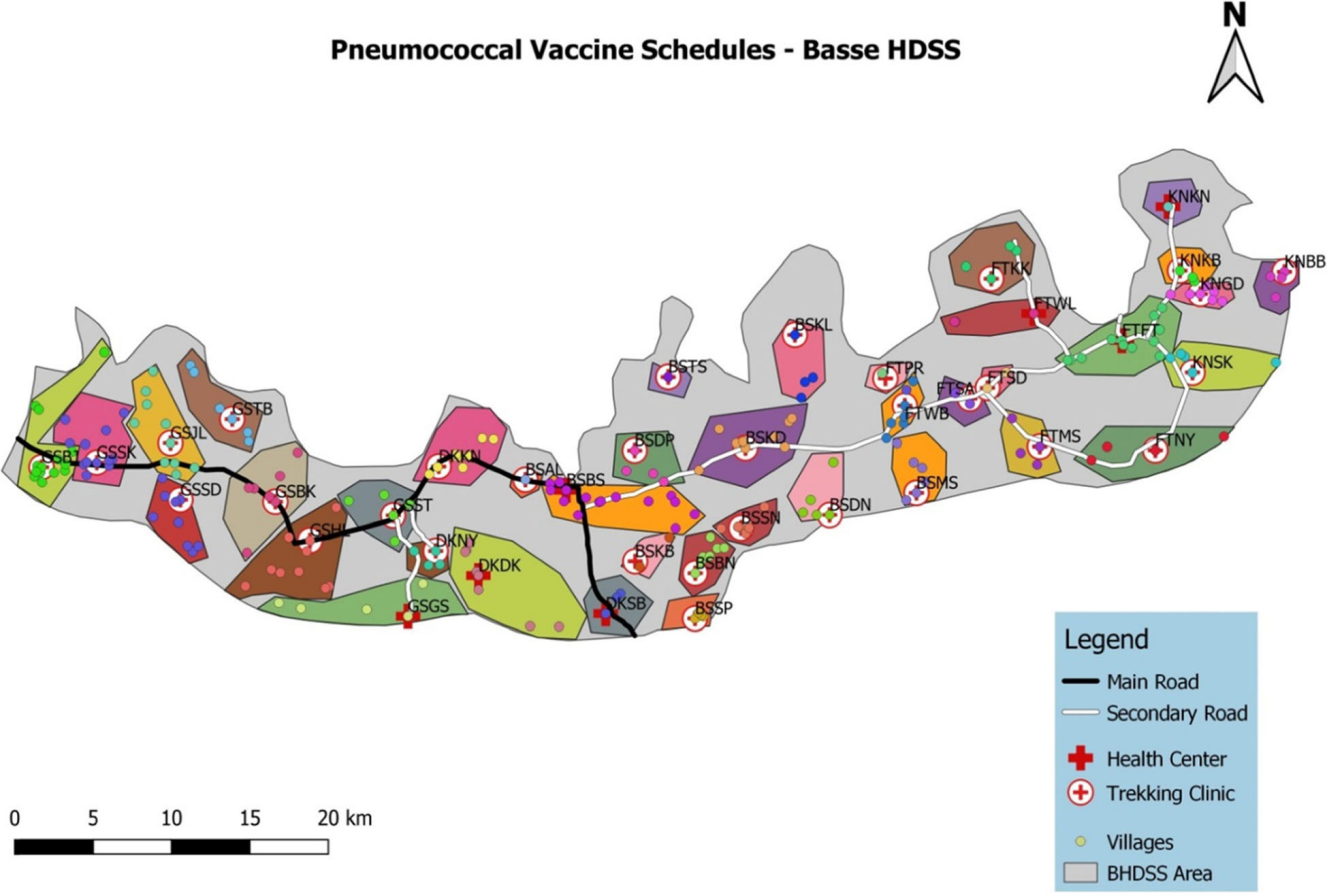
Map of the BHDSS showing 40 geographic clusters of villages assigned to one vaccination clinic

The PVS-AcqImm trial is being conducted in Upper River Region (URR) in the area covered by the BHDSS (Fig. 1). The trial will be based at the Basse Field Station of MRCG at LSHTM. The BHDSS population is 178,510 (225 villages) with 19% of the population aged < 5 years. The annual birth cohort is around 6000. The area has a child mortality rate around 50 per 1000 live births. There are 40 geographically separate clusters of villages assigned to attend geographically separate EPI clinics (Fig. 1). These geographic clusters of villages were randomly assigned to the 1+1 or 3+0 schedule in the PVS field trial. PVS-AcqImm participants will be selected from the 28 clusters closest to Basse town.

### Participant selection

The sampling frame for selection to receive the interventions will be all infants resident in the 28 selected clusters. Residency will be defined as:

• Born to or cared for by a parent or guardian who is resident for greater than 4 months as confirmed by BHDSS records or a household visit with the report of the household or compound head. Provisional residency may be established by the verbal report of the parent or guardian.

• Born to or cared for by a parent or guardian who intends to be resident for greater than 4 months with verification at a household visit and report of the household or compound head. Provisional residency may be established by the verbal report of the parent or guardian.

The sampling frame will be developed as part of the BHDSS in the trial area. Pregnancies and births in all households are registered from 4-monthly BHDSS enumerations of each household, village reporter records, and registration at EPI clinics. These data are electronically recorded in the field and synchronised centrally on a weekly basis. A verified and updated sampling frame is available for use each week. The sampling frame lists the mother’s name, infant’s name, date of birth, father’s name, name of household head, village name, compound number, and individual ID number.

All resident infants are eligible for enrolment in the large PVS field trial. Approximately 28 infants in each of the 28 clusters will be enrolled in the PVS-AcqImm trial with 784 participants for the study of pneumococcal carriage and 336 for measurements of immunogenicity (Table 2). Enrolment will proceed over a period of 4–10 months. A similar number of infants will be enrolled each month in each cluster selecting the first presenting infant each month and each sequential infant until the monthly target is achieved and approximately 28 participants are enrolled in each cluster. If 28 infants cannot be enrolled in all 28 clusters in a reasonable time, enrolments in ‘slow to recruit’ clusters may be distributed to other group-specific clusters resulting in some clusters having less or more than 28 participants.

**Table 2:** Trial groups and timeline of vaccination, specimen collection, and measurement of endpoints

	Group		
Age	3+0 Acquisition (*n* = 392) Immunogenicity (*n* = 112)	1+1 Acquisition (*n* = 196) Immunogenicity-YF/PCV co-administration (*n* = 112)	1+1 YF_**separate**_ Acquisition (*n* = 196) Immunogenicity-YF/PCV separate administration (*n* = 112)
**6 wk**	***PCV13*** ***NPS*** (*n* = 392)	***PCV13*** ***NPS*** (*n* = 196)	***PCV13*** ***NPS*** (*n* = 196)
**10 wk**	***PCV13***	***Blood*** IgG (*n* = 112) OPA (*n* = 60)	
**14 wk**	***PCV13***		
**18 wk**	***Blood*** IgG (*n* = 112) OPA (*n* = 60)		
**6 mo**	***NPS*** (*n* = 392)	***NPS*** (*n* = 196)	***NPS*** (*n* = 196)
**9 mo**	***YF*** ***Blood*** IgG (*n* = 112) ***NPS*** (*n* = 392)	***YF & PCV13*** ***Blood*** IgG (*n* = 56) ***NPS*** (*n* = 196)	***PCV13*** ***Blood*** IgG (*n* = 56) ***NPS*** (*n* = 196)
**10 mo**	***Blood*** YFNA (*n* = 112) IgG (*n* = 112) OPA (*n* = 60) ***NPS*** (*n* = 392)	***Blood*** YFNA (*n* = 112) IgG (*n* = 112) OPA (*n* = 60) ***NPS*** (*n* = 196)	***YF*** ***Blood*** IgG (*n* = 112) ***NPS*** (*n* = 196)
**11 mo**	***NPS*** (*n* = 392)	***NPS*** (*n* = 196)	***Blood*** YFNA (*n*=112) ***NPS*** (*n*=196)
**12 mo**	***NPS*** (*n* = 392)	***NPS*** (*n*=196)	***NPS*** (*n*=196)
**13 mo**	***NPS*** (*n* = 392)	***NPS*** (*n*=196)	***NPS*** (*n*=196)
**14 mo**	***NPS*** (*n* = 392)	***NPS*** (*n*=196)	***NPS*** (*n*=196)
**18 mo**	***Blood*** IgG (*n* = 112) OPA (*n* = 60) ***NPS*** (*n* = 392)	***Blood*** IgG (*n* = 112) OPA (*n* = 60) ***NPS*** (*n* = 196)	***NPS*** (*n*=196)

*PCV13* 13-valent pneumococcal conjugate vaccine, *YF* yellow fever vaccine, *IgG* serotype-specific anti-pneumococcal IgG, *YF NA* yellow fever neutralising antibody, *OPA* opsonophagocytic assay

### Eligibility criteria {10}

Participants must meet all of the inclusion criteria and none of the exclusion criteria to be eligible.

#### Inclusion criteria

• Resident in the study area

• Age 0–10 weeks

• Intention to reside in their residential cluster until 18 months of age

#### Exclusion criteria

• Intent to move out of the study area before 18 months of age

• Age greater than 10 weeks

• Prematurity < 34 weeks gestation

• Birth weight < 2.0 kg or weight < 2.5 kg

• History of invasive bacterial infection or measles

• Receiving long-term antibiotic therapy, i.e. greater than 4 weeks

• HIV infection in the infant or mother

• Chronic debilitating illness

• Immunosuppressive therapy or immunodeficiency disorder

• Contraindication to PCV13—severe hypersensitivity to a previous dose

• Contraindication to the YF vaccine

### Who will take informed consent? {26a}

Firstly, we informed the community leaders in the 28 selected clusters about the nature of the trial. Trained trial staff who speak the local languages presented the trial information and answered any questions.

Given that PVS-AcqImm enrols participants also enrolled in the PVS field trial, we obtain consent on one occasion for PVS and the follow-up and collection of specimens for PVS-AcqImm. As part of PVS procedures, we inform groups of parents or guardians of potentially eligible infants at immunisation clinics and other appropriate settings about the general nature of the PVS trial. Eligible infants making their first presentation to the immunisation clinic after birth are identified. Trial staff determine the parent or guardian’s first language and literacy. More than three-quarters of the adults in the trial area are not literate in English. Trained staff provide the trial information sheet to literate parents or guardians. If the parent or guardian is not literate, trial staff verbally explain standardised information concerning PVS and PVS-AcqImm as per the trial information sheet. Staff enquire whether there are any questions and seek informed consent. Mothers and guardians are encouraged to discuss participation with the infant’s father before giving consent. Trial staff address the questions and concerns of parents or guardians. Trial staff who speak the language of the parent or guardian conduct the informed consent process.

If the parent or guardian is illiterate, an impartial witness is present during the informed consent process. Each impartial witness receives the information sheet and consent form. Impartial witnesses are reimbursed according to the standard operating procedures of MRCG at LSHTM.

The consent of parents or guardians is recorded on a paper form. If literate in English, the participant’s parent or guardian signs and dates the consent form. If the parent or guardian is illiterate, the impartial witness attests to the participant’s understanding, that informed consent is freely given, and the responses to the specific questions on the consent form. The impartial witness marks the participant’s responses to each of the specific questions on the form. If the parent or guardian has understood the information, s/he thumbprint the consent form. The impartial witness signs the consent form and dates the participant’s thumbprint. If a guardian provides consent, this is documented on the consent form and a statement of guardianship is obtained, with the signature of a witness if the guardian is not literate in English. The staff member obtaining consent records their name and signature on the consent form and provides an identical copy to the parent or guardian. The person obtaining consent also provides a copy of the information sheet to the parent or guardian (including the free-call contact details of two trial staff).

### Consent for collection and use of participant data and biological specimens {26b}

Consent for the collection and use of participant data and biological specimens is specified in the trial information sheet and consent form. The consent form includes specific confirmation, marked on the form and entered in the database, confirming consent for the collection of specific numbers and types of specimens, future research using the specimens, shipping of specimens overseas, and use of unidentified data via MRCG authorised data repositories.

### Interventions

#### Explanation for the choice of comparators {6b}

Our choice to compare the 1+1 (doses of PCV scheduled at 6 weeks and 9 months of age) versus 3+0 (doses of PCV scheduled at 6, 10, and 14 weeks of age) schedules for PCV vaccination is based on the hypotheses that the 1+1 schedule will provide sufficient, but inferior, direct protection between 2 and 9 months of age, during which time the risk of VT disease in our setting is very low, and superior herd protection, and similar overall effectiveness compared to the 3+0 schedule. We also hypothesise that the 1+1 schedule will provide superior direct protection between 9 and 18 months of age compared to the 3+0 schedule. Finally, we chose to compare co-administration and separate administration of PCV and YF vaccines at 9 months of age as the YF vaccine is scheduled at 9 months of age in most African countries.

#### Intervention description {11a}

The experimental intervention in this trial is the scheduling of PCV13 for infants resident in geographic clusters of villages to receive the first dose due at 6 weeks of age and a booster dose scheduled at 9 months of age. The standard intervention is the scheduling of PCV13 for infants with doses due at 6, 10, and 14 weeks of age. A further intervention in the alternative schedule group is the scheduling in one group of the YF vaccine to be administered at 10 months of age and PCV13 at 9 months of age and in another group co-administration of PCV13 and YF vaccines at 9 months of age. Following informed consent, infants are registered on the trial and a trial identification sticker is fixed to their infant welfare card. At each visit, trial staff identify the infant and the group allocation, based on the trial sticker and residential location. Trial staff record whether infants are migrating, or intend to migrate, within or out of the trial area.

PCV13 (Prevnar 13®) vaccine, manufactured by Pfizer Ltd, is licenced in many countries and has been approved for use in The Gambia since 2011. Stabilised YF vaccine is manufactured by Institut Pasteur in Dakar, with WHO prequalification in 2001. The EPI procures these vaccines through global systems coordinated by UNICEF. This trial delivers PCV13 and YF vaccines in collaboration with, and through the structures of the Gambian EPI, and under the operational conditions of the public health system. The EPI receives PCV13 into a central cold storage facility. The vaccine is transported to the regional centres either by a specially designed EPI ‘cold van’ or by cold storage units carried by Regional Health Directorate (RHD) vehicles. The RHD in URR is based in Basse. The URR RHD stores vaccine in solar refrigerators in Basse. From Basse, small volumes of vaccines are distributed on a monthly basis to five ‘base clinics’ in Basse, Gambisara, Demba Kunda, Fatoto, and Sabi. These five base clinics administer vaccines in the 28 different geographic locations involved in PVS-AcqImm. Solar vaccine refrigerators are used for storage at the base clinics. The trial uses the existing EPI procedures to monitor and record the vaccine accountability and cold-chain documentation with daily logs.

Immunisation is undertaken at four of the fixed health centres in the study area on 1 or 2 days each week. Mobile clinics in the other 24 sites are held once or twice per month. Given the infrequency of EPI clinics, there is generally some delay in the time that vaccines are actually received. In 2016, coverage in the BHDSS of three doses of PCV at 12 months of age was 92% and coverage of one dose of measles vaccine was 82%.

### Criteria for discontinuing or modifying allocated interventions {11b}

Trial participants are discontinued from participation in the study if:

• Any clinically significant adverse event, intercurrent illness, or other medical condition or situation occurs such that continued participation in the study would not be in the best interest of the participant

• The parent or guardian so desires

Participants who attend an EPI clinic outside their cluster and within the study area, but continue to reside in their original cluster, receive the trial schedule originally indicated on their infant welfare card and on the trial sticker. If participants migrate within the study area before completing their PCV schedule, they continue to receive the trial schedule allocated in the cluster of their new residence. Participants who migrate after completing their PCV schedule do not receive any further doses of PCV in the cluster of their new residence. Parents in the 1+1 group are advised that if they migrate permanently out of the trial area they should attend the next available EPI clinic to complete the routine schedule for PCV. If an infant allocated to the 1+1 group visits an EPI clinic outside the study area, the parent is instructed to request that their child receive the trial schedule indicated on the infant welfare card and study sticker. The study sticker includes free-call telephone numbers so that parents, or EPI staff outside the study area, may call for guidance. Infants resident in the alternative schedule clusters who decline consent are assigned to the national standard schedule.

### Strategies to improve adherence to interventions {11c}

Adherence to the trial vaccination schedules is facilitated by exclusion criteria including an intention to migrate out of the study area in the next 18 months. Also, lists of infants allocated to the 3+0 group who have not completed three doses by the age of 5 months, and lists of infants allocated to the 1+ 1 group who have not received the booster dose by the age of 11 months, are generated every month to guide defaulter tracing at home visits throughout the study area.

### Relevant concomitant care permitted or prohibited during the trial {11d}

PCV13 has been co-administered with measles-mumps-rubella [23] and measles-mumps-rubella-varicella [24] vaccines, but the results of both these studies do not report investigations of potential interference between the vaccines. PCV10 (Synflorix®) has been co-administered with the YF vaccine in a study of an investigational GSK vaccine although results of investigations of potential interference between the vaccines have not been published. A different investigational PCV10 vaccine manufactured by the Serum Institute of India has been co-administered with the YF vaccine with non-inferior immune responses [25]. Studies of YF vaccine co-administration with polysaccharide protein-conjugate quadrivalent meningococcal vaccine have not detected any adverse interaction [26]. The investigators are not aware of any data, or ongoing studies, that evaluate potential immune interference with the co-administration of PCV13 and YF vaccines.

Participants admitted to the hospital with an acute medical problem will have a blood culture taken, a plasma aliquot stored, a rapid malaria test done, and haemoglobin concentration measured. Samples will not be collected from children admitted electively or those with surgical problems, trauma, acute burns, or non-infectious neonatal problems. Participants admitted with suspected sepsis defined according to standardised criteria will have a blood culture done and those with suspected meningitis will have a blood culture done and a lumbar puncture performed. For those admitted with clinical pneumonia, a danger sign, or focal chest signs, a blood culture and chest X-ray will be performed and pleural fluid or lung aspirate obtained as clinically indicated. Other investigations will be done according to the clinical judgement of the attending clinician.

### Provisions for post-trial care {30}

The trial may be stopped early if there is evidence that the risk of pneumococcal disease is greater in one compared to the other trial group. If the Data Monitoring Committee (DMC) recommends that the trial be stopped early, a joint meeting of the DMC, Trial Steering Committee (TSC), and Central Stakeholder Committee will make a recommendation to the Sponsor regarding post-trial procedures, including whether a dose of PCV be administered to children in a group found to be inferior. LSHTM carries clinical trial/non-negligent harm insurance and medical malpractice insurance applicable to this trial.

### Outcomes {12}

The primary outcome of the immunogenicity study is serotype-specific IgG for VT at 18 months of age, analysed as the ratio of the geometric mean concentrations (GMC) in 1+1 compared to 3+0 participants. If serotype-specific IgG concentrations at 18 months of age are greater in the 1+1 compared to the 3+0 age group, we will infer that the schedule including the PCV booster dose provides a superior immune response to the standard schedule at 18 months of age. This superior immune response may be associated with reduced acquisition of VT colonisation and reduced risk of VT disease in the second year of life.

The primary outcome of the acquisition study is the rate of NP VT acquisition from 9 to 14 months of age in 1+1 compared to 3+0 schedule participants. The rate of acquisition will be the number of VT pneumococcal carriage acquisitions detected in six monthly NP specimens collected between the ages of 9 and 14 months. If the rate of VT acquisition is reduced in 1+1 compared to 3+0 participants at this age, we will conclude that the PCV booster dose provides superior protection from acquisition at this age with the implication of reduced transmission and potentially greater herd protection.

The primary outcome of the investigation of co-administration of PCV13 and YF vaccines is the proportion of participants with YF neutralising antibody titres of ≥1:8 comparing those who receive the vaccines separately or co-administered. If the difference in proportions in the two groups is non-inferior, we will conclude that there is no immune interference when the vaccines are co-administered. This finding would support a policy of co-administration of PCV13 and YF vaccines.

Secondary immunogenicity outcomes include comparison in alternative versus standard schedule groups, of:

- Pneumococcal VT opsonophagocytic activity (OPA) following a single dose at age 6 weeks, following three primary doses, following the booster dose at age 9 months, and at 18 months of age
- The proportion of participants with GMC of pneumococcal VT IgGs ≥0.35 μg/ml, 4 weeks after the primary series, 4 weeks after the booster dose at age 9 months, and at 18 months of age
- Geometric mean concentrations of pneumococcal VT IgGs 4 weeks after administration of PCV13 at 9 months of age, with and without co-administration with the YF vaccine

These secondary immunogenicity outcomes will demonstrate the functionality of anti-pneumococcal IgG antibodies throughout the different schedules, which is required when investigating PCVs [27]. In addition, we will investigate the potential effect on PCV13 immunity of co-administration with the YF vaccine, and so provide additional data on the safety of co-administration.

Secondary acquisition outcomes include the:

• Rate of NP acquisition of non-VT pneumococci between 9 and 14 months of age

• The proportion or participants with NP VT colonisation at 6, 9, and 18 months of age

These secondary outcomes will provide information on the potential for the PCV13 booster dose to increase the rate of non-VT pneumococcal acquisition and the comparative effects of the two schedules on VT prevalence at different age points.

### Participant timeline {13}

**Fig. 2:**
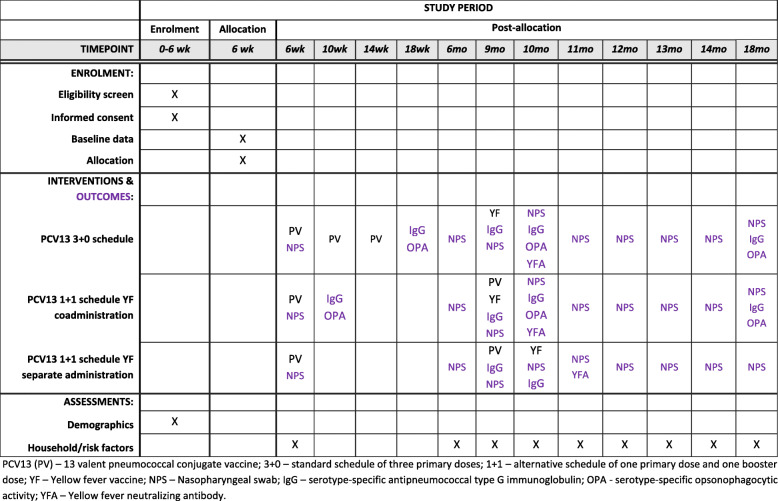
Schedule of enrolment, interventions, and assessments. *PCV13 (PV)*, 13-valent pneumococcal conjugate vaccine; *3+0*, standard schedule of three primary doses; *1+1*, alternative schedule of one primary dose and one booster dose; *YF*, yellow fever vaccine; *NPS*, nasopharyngeal swab; *IgG*, serotype-specific anti-pneumococcal type G immunoglobulin; *OPA*, serotype-specific opsonophagocytic activity; *YFA*, yellow fever neutralising antibody

Participants in the trial are enrolled in one of three different groups with administration of intervention PCV13 and YF vaccine schedules as shown in Table 2 which also shows the timing of specimen collection and measurement of endpoints. The SPIRIT figure (Fig. 2) shows further details of the participant schedule for enrolment, interventions, and assessment.

### Sample size {14}

For measurement of the effect of the PCV booster dose on the rate of acquisition of VT pneumococci, the smallest clinically important hazard ratio to detect is 0.75. A Dutch study of VT colonisation following a PCV booster dose at 12 months of age compared to no booster showed a relative risk reduction of 0.64 [14]. Thus, the hazard ratio of 0.75 is a more conservative estimate of the smallest relevant clinical effect size. Serotype-specific anti-pneumococcal IgG to the serotypes included in PCV13 will be analysed as a fold difference in GMCs as our interest is to test whether antibody concentrations are different and not necessarily correlates of protection. We define superiority as a 1.8-fold or greater difference in GMC for ≥10/13 serotypes, an approach used in other similar studies [28]. The 1.8-fold difference is based on data comparing IgG concentrations in Vietnamese children receiving PCV10 schedules of 3+1 or 3+0 in whom GMCs 1 month following the booster dose were 3-fold or more greater for all VT (pers. comm. P Licciardi 23 Apr 2018). Non-inferiority of response to the YF vaccine will be defined as the lower limit of the 95% confidence interval for the absolute difference in proportions with neutralising titres of YF antibody ≥1.8 being greater than −10% [26, 29].

The current prevalence of NP VT pneumococcal colonisation in those aged less than 2 years in the study area is 17% (author’s own data). This measurement used selection of one or more morphologically different colonies, while the latex sweep serotyping method used in this study is more sensitive to multiple serotype colonisation so we assume a slightly higher prevalence. The assumptions included in the sample size calculations are that the baseline risk of VT colonisation at any one point in time is 20%, participant withdrawal of 2%, death rate of 2%, loss to follow-up of 3%, a 4% rate of cross-over between clusters, and 35% censoring of participants who do not experience VT colonisation. We have used local baseline data on the variability of cluster-specific pneumococcal carriage to calculate an intra-cluster correlation of 0.02. The probability that VT pneumococci will be detected in the five NP swabs collected between 10 and 14 months of age in the 3+0 group is 1–0.80^5^; that is, the expected proportion of participants who acquire VT pneumococci is 0.67. Using a survival analysis logrank test and assuming a 5% level for statistical significance and 80% power, the study requires 784 participants with 28 participants in 14 clusters in each group to detect a hazard ratio of 0.75. The sample size was inflated from 392 to 532 with the intra-cluster correlation factor and then to 784 when taking into account rates of death, loss-to-follow-up, withdrawal, cross-over, and censoring due to no detection of vaccine-type colonisation.

The immunogenicity study includes two primary endpoints and the sample size calculation for each used *α* = 0.025 and *β* = 0.9. For the first primary endpoint, our interest is in the relative magnitude of the IgG response in the two groups. Thus, we will test the alternative hypothesis of a ≥1.8-fold difference in GMCs between the 1+1 and 3+0 groups for ≥10/13 serotypes at 18 months of age. Baseline GMCs and their standard deviations for each serotype in the 3+0 group were taken from findings in the USA [30] and Fiji [31]. Non-independence of observations within clusters was taken into account in the following way; given 112 participants in each group with 5% loss to follow-up (i.e. 106 participants per group) and eight participants in each of 14 clusters, and a variance inflation factor = 1 + (*m* − 1) × ICC = 1 + (8 − 1) × 0.01, the effective sample size in each group is *n*/VIF = 106/(1 + (8 − 1) × 0.01) = 99. Based on local cluster-wise variation in the mean prevalence of VT colonisation, the intra-cluster correlation = 0.01. Thus, simulations assumed 99 participants per group. Data were simulated so that there was a 1.8-fold difference in the IgG levels in the two groups, and differences in serotype-specific GMCs were tested using a *t*-test. Within-subject correlation between the multiple serotype-specific endpoints was captured by a subject-level variation term with standard deviation 0.4 in a random-effects linear regression model. Superiority was declared for the overall conclusion if at least 10 out of 13 tests rejected the null hypothesis in 5000 simulations. Table 3 shows the results of the power calculations indicating power for the overall conclusion of superiority is 99%.

**Table 3:** Results of power calculations to test for 1.8-fold differences in geometric mean concentrations of serotype-specific IgG concentrations

Power for overall conclusion (1.8-fold difference)		99%
Power for serotype-specific conclusions	1	> 99%
	3	> 99%
	4	93%
	5	93%
	6A	86%
	6B	95%
	7F	> 99%
	9V	> 99%
	14	98%
	18C	97%
	19A	93%
	19F	92%
	23F	92%

For the second primary endpoint, power was calculated using a formula for cluster-randomised non-inferiority study using Nquery + nTerim software. Calculations assumed that 95% of participants had YF neutralising antibody titres ≥ 1:8, [29] *ICC* = 0.01, and seven or eight participants per cluster (seven to account for loss to follow-up). Power to detect a 10% absolute difference in proportions with seven participants per cluster was 86% and 90% with eight participants per cluster. With the inclusion of 28 clusters and seven or eight participants per cluster, this leads to a sample size of 196 or 224 participants.

### Recruitment {15}

All resident infants will be eligible for enrolment in the PVS field trial, which enrols approximately 150 participants per week. PVS participants may also be enrolled in PVS-AcqImm; 28 infants in each of the 28 clusters will be enrolled with a total of 784 participants for repeated measures of pneumococcal colonisation. Of the 392 participants resident in the 1+ 1 clusters, a sub-sample of 224 will be enrolled for measurement of immunogenicity endpoints, 16 in each of the 14 clusters. Of the 16 infants targeted in each 1+1 cluster, a target of eight will be allocated to 1+1 with PCV13–YF vaccine co-administration and a target of eight allocated to separate administration, i.e. 112 infants will be allocated to each of the co-administration and separate administration groups (Table 2). Of the 392 participants resident in the 3+0 clusters, a sub-sample of 112 will be enrolled for the measurement of immunogenicity endpoints.

A similar number of infants will be enrolled each month in each cluster for approximately 10 months selecting the first presenting infant each month and each sequential infant until the monthly target is achieved. For repeated measures of pneumococcal colonisation, enrolment will be implemented by selecting the first presenting infants in each cluster in each month until the target of 28 participants are enrolled in each cluster (less or more than 28 may be enrolled per cluster). In the 1+1 clusters, infants will be allocated to PCV13–YF vaccine co-administration or separate administration groups according to a prepared random allocation list including 14 clusters with a target of 16 infants (less or more than 16 may be enrolled per cluster) in each cluster. In each of the 3+0 clusters, a target of eight infants will be enrolled in each cluster for measure measurement of immunogenicity endpoints, i.e. a total of 112 participants. If insufficient numbers of participants are enrolled in a cluster in 1 month, then additional participants may be enrolled in that cluster in the following month. If a participant withdraws or is lost to follow-up, additional participants may be enrolled in both groups to ensure sufficient enrolment and the use of identical procedures in the two groups.

### Assignment of interventions: allocation

#### Sequence generation {16a}

Sixty-eight PVS trial clusters were randomised using a blocked scheme to ensure similar numbers of clusters were assigned to each group. Randomisation was stratified by a binary variable correlated with ‘high’ or ‘low’ cluster-level incidence of clinical pneumonia. Randomisation was carried out in permutations using the above stratification until selections achieved balance in terms of the presence of a health centre and balance on population size between the two groups. In order to select the stratifying variable of cluster-level incidence of clinical pneumonia, the cluster-level prevalence of ‘high’ or ‘low’ VT carriage in children with clinical pneumonia in the BHDSS was correlated with cluster-wise population density, rates of hospitalisation, clinical pneumonia, radiological pneumonia, IPD, and mortality. Of the five listed outcomes, clinical pneumonia incidence had the closest correlation with VT carriage prevalence and thus was chosen as the stratifying variable.

Individuals selected for participation in PVS-AcqImm will be selected from the 28 clusters closest to Basse (Fig. 1). Of these 28 clusters, 14 are allocated to each of the 1+1 and 3+0 groups, four of these 28 clusters include health facilities (two in the 1+1 group), and 14 are stratified as high clinical pneumonia incidence (seven in the 1+1 group). Thus, the clusters selected for PVS-AcqImm are appropriately balanced for group allocation, clinical pneumonia incidence, and the presence of a health facility.

Pre-prepared random assignment lists, by cluster, are used at the time of enrolment to determine participant assignment to different schedules of blood collection. In the 1+1 clusters, the pre-prepared random assignment lists specify assignment to the PCV–YF vaccine co-administration or separate administration groups.

#### Concealment mechanism {16b}

A public event was held to announce group allocations of each cluster of villages in the PVS trial area. Representatives of each cluster were present at the public event. Selection of the randomisation list at the public event involved random selection of one of 100 valid randomisation lists. Thus, the investigators and cluster representatives had no knowledge of the allocation sequence at the time of group allocation.

#### Implementation {16c}

An independent statistician prepared the cluster randomisation lists. Trial staff enrol the participants. The trial data manager produced the lists for random assignment of participants to different schedules of blood collection and co-administration or separation administration of PCV–YF vaccines.

### Assignment of interventions: blinding

#### Who will be blinded {17a}

Vaccinators and parents will be aware of the schedules used. Laboratory staff will be blinded as specimens will be labelled with a unique identification number that does not identify the study group. Blinding of laboratory staff will avoid bias given the laboratory-based objectives of the study. Statisticians will analyse date in a pseudo-blinded fashion with the two groups identified by an indicator label rather than the identity of each group.

#### Procedure for unblinding {17b}

Given that participants are not blinded to their group allocation, a procedure for unblinding is not needed.

### Data collection and management

#### Plans for assessment and collection of outcomes {18a}

Participants are under passive surveillance for clinical events as per the procedures of the PVS field trial. If participants present to a health facility in the trial area, staff will provide standardised evaluation, investigation, and recording of clinical safety events in an electronic medical record (see the “Relevant concomitant care permitted or prohibited during the trial {11d}” section). Details of the surveillance for clinical safety events are provided in an accompanying protocol paper for the PVS field trial (manuscript submitted to *Trials*, TRLS-D-21-01145).

Selection bias is limited by pre-selection of infants within cluster to specified schedules of specimen collection. Baseline demographic and clinical information is recorded with a questionnaire administered at each visit documenting intercurrent illnesses and factors that may influence pneumococcal colonisation. Data collection forms are not included in the protocol but are available on request.

The allowable window period for NP specimen collection is 14 days or more between swabs with the age 14 months specimen being collected no later than 15 months of age. Trained staff collect NP specimens according to WHO recommendations [32]. Biased case ascertainment is minimised by continuous quality control for the technique of NP specimen collection and allowing only a minimum number of staff collecting NP specimens. The allowable window period of blood collection will be within 35 days of the target date.

An event of VT colonisation will be defined as detection of a pneumococcus in a NP specimen belonging to serotypes 1, 3, 4, 5, 6A, 6B, 7F, 9 V, 14, 18C, 19A, 19F, or 23F, using latex sweep methods. All other serotypes will be defined as non-VT. Non-typeable isolates will be defined as pneumococcal by colony morphology and biochemical means, not serotypeable by latex sweep or Quellung methods. An event of pneumococcal acquisition will be defined as detection of a pneumococcal serotype in a NP specimen that was not detected in the previous NP specimen.

#### Plans to promote participant retention and complete follow-up {18b}

Participants are only eligible for enrolment if intending to reside in the trial area until 18 months of age. The informed consent process allows time for discussion among family members, and we specifically aim to discuss consent with the father of the child. We provide participants with a trial information sheet that includes free-call phone numbers that can be used at any time. Trial information specific to every individual participant is provided in the form of a pictorial guide to the follow-up timeline and specimen collection schedule. Trial staff use the phone numbers of parents to communicate and facilitate complete follow-up. We provide instructions to participants if attending an EPI clinic outside the trial area that facilitates administration of vaccines according to the trial schedule.

#### Data management {19}

A data management plan has been prepared and approved by the MRCG Head of Data Management and is available on request. Data are collected on eCRFs using a standardised format. Electronic data capture (EDC) is done offline using encrypted devices which is then synchronised with a central server on a weekly basis. Trial staff attend all EPI clinics, confirming the identity of all infants and record immunisation data in real time. Trial staff generate source data on electronic devices at regular visits as per the trial timeline.

Data entered into eCRFs will be monitored for completeness and consistency against the relevant source documents. Independent trial monitors undertake 100% verification of source data for the primary endpoints and informed consent. Anomalies identified are reconciled with the source.

Front-end data quality checks are programmed into the data capture application. Backend edit checks and validation checks are built-in to monitor the validity of data (e.g. to identify inconsistent dates and times, and clinical and antropometric measurements outside defined ranges, etc.). Data queries are generated weekly by the data manager for resolution by trial staff. Reports of data quality are generated periodically.

The trial database is housed on a secure network server with restricted access to the backend. The backend comprises a MSSQL database which will be regularly backed up as part of the organisation’s disaster recovery plan. An in-house Web-based application is used for the database structure, using the PHP\ASP.Net platform connecting to the MSSQL backend database. A recovery point objective is set so that systems and data will be restored back to their prior status 24 h prior to a failure. Daily and monthly backups of all media servers are done to achieve this objective.

The e-CRF is used as the specification for the design of the database. An annotated CRF indicates the relationship between the variable names in the database and the fields in the CRF. A data dictionary uses a standard template and includes a list of database variable names, variable descriptions, data types, and sources, valid values, and in-built edit checks. Standard data coding will be used (e.g. latest MedDRA version accessed through the Internet will be used for adverse event reporting).

The long-term storage of research records is done in accordance with MRCG policies and procedures for archiving. All paper records will be held for at least 10 years in the Unit’s archiving facility. Data in electronic format will be held indefinitely on our Electronic Data Repository. The trial is run in compliance with the MRC Corporate Information Security Policy [33] and the Unit’s Information and Communication Technology Security Policy. The study is conducted in compliance with the ICH Harmonized Tripartite Guideline for Good Clinical Practice E6 (R2 Step 4).

#### Confidentiality {27}

No subject identifiable information (names, addresses, etc.) is entered into the database. Identifiable information is stored in the electronic medical record, vaccination and BHDSS databases, encrypted and password-protected, and accessible to only a limited number of staff involved in the care of patients. Trial monitors only access pre-specified, non-identifiable data. Informed consent documents are stored in locked, fire-resistant filing cabinets to which only the Principal Investigator and a limited number of delegated clinical trials personnel have access. Data are backed up at the end of each day in the field on encrypted flash drives to prevent any data loss during transit. All computers within MRCG are access controlled with strong password policies that prevent unauthorised access to networked user machines. Users to whom network access has been given are granted necessary privileges to the trial database based on their trial roles.

#### Plans for collection, laboratory evaluation, and storage of biological specimens for genetic or molecular analysis in this trial/future use {33}

The trial collects blood and NP specimens but procedures do not include genetic or molecular analyses of human material.

Flocked nylon swabs are placed in media and transported to the MRCG laboratory in Basse within 6 h or placed in a dry shipper for later transport to Basse. NP specimens are processed in the Basse laboratory. A 10-μl loop of vortexed NP specimen is inoculated onto blood agar with 5% gentamicin and incubated in CO_2_. Identification of pneumococcal colonies follows recommended methods [32]. Specimens positive for the pneumococcus undergo latex sweep serotyping in Basse. Morphologically different pneumococcal colonies are selected from the primary plate, purified, and stored. For internal quality control (QC), a proportion of NP specimens is processed by two different operators and results compared. External QC on sweep serotyping involves blind assessment of known spiked samples, prepared by the Murdoch Children’s Research Institute (MCRI) Pneumococcal Laboratory, placed among the routine specimens delivered to the laboratory. As further validation of the latex sweep serotyping, a proportion of the specimens that are positive for pneumococcus will be subjected to serotyping by microarray at BUGS Bioscience - St George’s University of London. Microarray is the most sensitive method for detecting carriage of multiple pneumococcal serotypes; its specificity is similar to high-quality latex sweep [34]. The Basse laboratory undergoes external quality control for bacteriology according to One World Accuracy International (Burnaby, British Columbia, Canada).

Blood specimens collected in serum separation tubes are transported in a cool container to the Basse laboratory within 6 h, centrifuged, and aliquoted. Aliquots are stored in Basse at −70 °C prior to shipment to MCRI for pneumococcal serology and to the Bernard Nocht Institute for Tropical Medicine (BNITM), Hamburg, for YF serology.

Serotype-specific anti-pneumococcal IgG and OPA will be measured in the Pneumococcal Laboratory at MCRI. A standardised ELISA will be used. Microtitre wells are coated with pneumococcal polysaccharide, depending on the serotype. To neutralise cell wall polysaccharide antibodies, diluted serum samples are incubated with cell wall polysaccharide and polysaccharide of serotype 22F, before further dilutions. A reference serum (007sp, Food and Drug Administration, Bethesda MD) is used and incubated with cell wall polysaccharide and 22F polysaccharide. Horseradish peroxidase-conjugated anti-human IgG and the TMB Peroxidase Substrate system is used for detection. Three control sera are used to assess inter-assay variation. A standardised and validated OPA [35] will measure the serotype-specific opsonophagocytic and killing activity of anti-pneumococcal IgG.

Neutralising antibodies against the YF virus will be assayed at BNITM. Neutralisation titres will be expressed as the serum dilution yielding ≥50% neutralisation (i.e. in which one or both of two duplicate standard cellular viral infections is blocked). If a complete infection is observed at all serum dilutions, the neutralisation titre is recorded as < 1:4. Seroprotection is defined as a neutralisation titre ≥1:8.

### Statistical methods

#### Statistical methods for primary and secondary outcomes {20a}

The primary analysis of the effect of the PCV13 booster dose on pneumococcal acquisition will use data from the six NP specimens collected between 10 and 14 months of age. Detection of homologous serotypes within an individual on multiple occasions will not be counted as multiple acquisitions. That is, the maximum number of acquisitions for one participant will be five. Analysis will employ a Cox proportional hazards regression model comparing the hazard ratio for VT acquisition in the 1+1 compared to the 3+0 group including variables to adjust for previous serotype-specific acquisition and trial cluster. The interpretation of the models will be at the individual level.

Secondary analyses of the endpoint of non-VT acquisition will use the same approach as described above. For the endpoint of VT prevalence at 6, 9, and 18 months of age, we will compare proportions while also taking into account the trial cluster.

Analyses of immunogenicity data will be per-protocol for non-inferiority endpoints and otherwise by modified intention-to-treat with all individuals included by randomised group if they have a laboratory result. For the primary endpoint, we will compare the VT serotype-specific GMCs in the two groups using linear regression including co-variates of baseline IgG, cluster, and season of PCV13 booster if associated with GMC, using separate models for each serotype. For calculation of GMCs, a logarithmic transformation will be applied to the IgG concentration value prior to analysis. The 1+1 schedule response will be deemed superior if the ratio of GMCs is ≥1.8 for ≥10/13 serotypes at the 5% level of significance.

Proportions of participants with YF neutralising antibody titre ≥1:8 will be calculated with exact binomial 95% confidence intervals (CI). The CI for the difference in the proportions will be computed using the Miettinen-Nurminen method (or another appropriate method), including cluster and/or season as needed. If the lower limit of the CI for the difference in proportions is greater than −10%, response in the co-administration group will be deemed non-inferior.

#### Interim analyses {21b}

There are no planned interim analyses.

#### Methods for additional analyses (e.g. subgroup analyses) {20b}

An exploratory analysis will include repeated acquisitions of homologous serotypes, that is counting repeated acquisitions separately, testing whether the rate of individual VT acquisitions, including repeated acquisitions, is reduced in the 1+1 compared to the 3+0 group.

#### Analysis methods to handle non-adherence or missing data {20c}

The protocol specifies enrolment of additional participants if a participant is lost to follow-up. Analysis will handle events of non-adherence by restricting the data included based on certain conditions. Receipt of the third dose of PCV13 in the 3+0 group at greater than 23 weeks of age will be considered outside the eligible window for per-protocol analysis. Receipt of the booster dose of PCV13 in the 1+1 group at greater than 11.5 months of age will be considered outside the eligible window for per-protocol analysis. Receipt of the YF vaccine in the 1+1 YF co-administration group at greater than 11.5 months of age will be considered outside the eligible window for per-protocol analysis. Receipt of the YF vaccine at greater than 12.5 months of age by infants allocated to separate administration of PCV13 and YF vaccines will be considered outside the eligible window for per-protocol analysis. Inclusion of the age 18 months blood specimen in the per-protocol analysis will require collection no later than 20 months of age. Analyses will be conducted with the available data.

#### Plans to give access to the full protocol, participant-level data, and statistical code {31c}

The protocol is available on request. The data generated will be suitable for sharing in an anonymised format. The data will be in CDISC ODM format that is an internationally recognised standard. Data will not be deposited into a central repository but held securely by MRCG. The trial is registered with the International Standard Clinical Trial Registry Network (ISCTRN) to maximise its visibility to other interested parties. Summary data will be provided through ISCTRN at the end of the study. Data sharing will follow MRCG policy [36]. Applications for access to the complete datasets will need to be made to the MRCG Unit’s Archives department who will then forward it to the Scientific Coordinating Committee (SCC) of the Unit. All requests for the dataset will be reviewed by the SCC and also by The Gambia Government/MRC Joint Ethics Committee (GG/MRCG JEC) to establish that the request is in order to perform scientifically appropriate analysis. The datasets collected within the trial will be available to other users once all relevant trial-related publications in scientific journals have been accepted. Prior to this point, requests will be considered on a case by case basis.

- For practical reasons, this time period for trial-related publications may be indicative and might need to be revised if delays occur. Different periods may be applied to different datasets, e.g. to take account the complexity of cleaning and documentation.
- Timing will depend on the trial’s data collection patterns.
- In relation to timing, the terms could, for instance, be expressed as follows: ‘6-months after the end of the current grant period’, ‘12-months after new data collection to allow for data cleaning and documentation’, or ‘3-months following the first publication of findings based on the data’.

Statistical code will be available on request.

### Oversight and monitoring

#### Composition of the coordinating centre and trial steering committee {5d}

The trial management group meets every week and includes the Principal Investigator, Trial Epidemiologist, Trial Coordinator, Project Manager, Data Manager, and Higher Laboratory Scientific Officer. The Trial Steering Committee (TSC) is composed of a Chairperson, an expert clinician, an expert trialist, an expert laboratory scientist, the national EPI Programme Manager, members of the URR and CRR RHDs, two community representatives, and a Sponsor representative.

#### Composition of the data monitoring committee, its role, and reporting structure {21a}

The DMC is independent of the Sponsor and composed of a Chairperson, expert clinician, expert statistician, and an independent statistician. The role of the DMC is described in its Charter (see Supplementary material) and is to protect and serve trial participants and to assist and advise the PI so as to protect the validity and credibility of the trial. The DMC monitors the safety of the trial; reviews its progress and accruing data; makes recommendations to the TSC whether the trial should continue, be terminated, or modified; and determines if interim analyses should be undertaken. The DMC also considers data quality, recruitment, compliance with the protocol, sample size assumptions, data emerging from other related studies, requests for interim trial data, and the final data and its interpretation. Materials and discussion during meetings are confidential. The DMC meets every 4–6 months.

#### Adverse event reporting and harms {22}

Adverse events are defined according to the ICH Harmonised Guideline for GCP E6(R2). Due to the established safety record of PCV13 and YF vaccines, the trial does not record solicited events of reactogenicity. The trial uses an electronic vaccine record system to record unsolicited events of reactogenicity when reported by caregivers within 7 days of a dose of PCV13. Unsolicited events of reactogenicity following a dose of the YF vaccine are recorded up to 1 month post-administration. Serious adverse events (SAEs) are tabulated in reports every 3 months. Severe adverse drug reactions (SADRs) and serious, severe unexpected serious adverse reactions (SUSARs) are reported to the Sponsor within 24 h. The Sponsor reports SADRs and SUSARs to the Gambia Medicines Control Agency (GMCA), while PI reports these events to the Gambia Government/MRCG Joint Ethics Committee, LSHTM Ethics Committee, TSC, and DMC.

Follow-up and resolution of SAEs are recorded in electronic reports. The Sponsor, TSC, and DMC are informed of SAEs at each meeting. The MRCG Clinical Trials Department Coordinator reports SAEs to the GMCA. The PI reports SAEs to the TSC and DMC. SADRs and SUSARs will be reported to the GG/MRC JEC within 5 working days. Deaths unrelated to the intervention are reported to the GG/MRC JEC at the next meeting. Information on unanticipated changes that may increase the risk to participants or may affect adversely the safety of the participants or the conduct of the trial or that could alter the EC’s approval to continue the trial will be reported to the Sponsor, GG/MRC JEC, LSHTM EC, GMCA, TSC, and DMC in writing within 2 working days.

#### Frequency and plans for auditing trial conduct {23}

The GG/MRCG JEC may audit the conduct of the trial at any time, independent of the investigators and Sponsor. The trial management group meets weekly to review progress in recruitment, clinical endpoint surveillance, and data quality. The trial management group meets monthly to review standardised indicators of quality assurance of all study procedures and complaints. The TSC meets 1 to 2 months after every DMC meeting, which occur approximately every 4–6 months. The TSC sets targets for recruitment, data collection, and protocol compliance. All trial-related complaints are reviewed by the TSC. The trial statistical analysis plan will be submitted for approval by the TSC. The TSC considers new information relevant to the trial, including reports from the DMC and the results of other studies that may have a direct bearing on the future conduct of the trial. Annual reports are submitted to the GG/MRCG JEC and LSHTM EC which document progress in recruitment, SAEs, and protocol deviations and violations.

#### Plans for communicating important protocol amendments to relevant parties (e.g. trial participants, ethical committees) {25}

Protocol amendments are reported to the Sponsor, trial monitors, SCC, GG/MRCG JEC, LSHTM EC, GMCA, DMC, TSC, and clinical trial registry (ISRCTN). Deviations from the protocol are fully documented using a non-adherence/compliance report form.

#### Dissemination plans {31a}

The investigators intend to publish the results of the trial in peer-reviewed scientific journals. All trial publications will follow MRC guidelines for Open Access publishing. The results of the study will be disseminated to the parents/guardians of each participant and the communities in the study area. The findings of the trial will be presented to the Central Stakeholders Committee that includes representatives of the Ministry of Health EPI and other regional and central health authorities. The results of the trial will be presented to the WHO EPI department and its Strategic Advisory Group of Experts on Immunisation.

## Discussion

The start of recruitment in the trial was delayed in 2020 due to restrictions related to the COVID-19 pandemic. Since the beginning of recruitment, we have not experienced any significant interruptions to trial implementation.

## Trial status

The current protocol version is 5.0, dated 24 May 2021. Recruitment began on 14 September 2020. Recruitment was completed on 28 October 2021.

## Supplementary Information

**Additional file 1:.** Model Informed Consent form.

## Abbreviations

BHDSS: Basse Health and Demographic Surveillance System
CRF: Case report form
CRR: Central River Region
DMC: Data Monitoring Committee
EC: Ethics Committee
EPI: Expanded programme on immunisation
GCP: Good Clinical Practice
GG: Gambia Government
GG/MRCG JEC: Gambia Government/Medical Research Council Unit The Gambia Joint Ethics Committee
GMCA: The Gambia Medicines Control Agency
ICH: International Conference on Harmonization
IgG: Immunoglobulin G
IPD: Invasive pneumococcal disease
LSHTM: London School of Hygiene & Tropical Medicine
LSHTM EC: London School of Hygiene & Tropical Medicine Ethics Committee
MCRI: Murdoch Children’s Research Institute, Melbourne
MoH: Ministry of Health
MRCG: Medical Research Council Unit The Gambia
NP: Nasopharyngeal
PCV: Pneumococcal conjugate vaccine
PI: Principal Investigator
PSP: Pneumococcal Surveillance Project
PVS: Pneumococcal Vaccine Schedules study
SAE: Serious adverse event
SCC: Scientific Coordinating Committee
SUSAR: Serious unexpected serious adverse reaction
TSC: Trial Steering Committee
URR: Upper River Region
VT: Vaccine-type
WHO: World Health Organisation
YF: Yellow fever
