# Supplementary material for: Pneumococcal conjugate vaccination schedules in infants—acquisition, immunogenicity, and pneumococcal conjugate and yellow fever vaccine co-administration study
Source: Trials. 2022 Jan 15;23:39. doi: 10.1186/s13063-021-05949-4 (PMC8760872; doi:10.1186/s13063-021-05949-4)
Supplement: Supplementary file 1 — Additional file 1:. Model Informed Consent form. [file 13063_2021_5949_MOESM1_ESM.docx]

**PARTICIPANT INFORMATION SHEET**

**MRCG at LSHTM/Gambia MoH - Pneumococcal Vaccine Schedules**

**Acquisition/Immunogenicity/Co-administration – 1+1 YF Separate Group**

Version 4.0, approved 17 Feb 2021, SCC number 1670.

The Sponsor is LSHTM. The funders are MRC, Wellcome, UKAID, NIHR and Bill & Melinda Gates Foundation.

You are invited to let your child take part in a research study. You should understand how and why the study is being done. You can ask questions at any time. You may wish to speak with your spouse or family members before deciding to let your child take part. If you allow your child to join the study, you will need to sign or thumbprint a consent form, which shows that MRC at LSHTM obtained your agreement.

The study will test whether two different schedules of pneumococcal vaccine have the same impact. The usual schedule includes doses at 6, 10 and 14 weeks of age. The alternative schedule includes doses at 6 weeks and 9 months of age. Similar studies are underway in Vietnam, India and South Africa.

The benefits of the alternative schedule are: a) less cost to governments and the savings may be used to introduce other vaccines, b) less cost to donors and the savings may be used to support other vaccines in low-income countries, c) two less injections early in life, d) less pain, e) greater flexibility, acceptability and sustainability of immunisation programmes.

The study will collaborate with the GG and operate south of the river from Jakhaly to Koina. The study will use the current vaccine that is used throughout the country. Half of the immunisation clinics will continue to deliver the current schedule and half will change to the alternative schedule. Your village and child had a 50:50 chance of being allocated to the alternative or standard schedule. All newborns will be eligible for the study. The study will begin in 2019 and go for 4 years.

Staff in health facilities will identify children with suspected meningitis, blood infection or pneumonia and work with GG staff to provide standard diagnosis and treatment. Nasal samples will be collected from sick children at health facilities and infants at immunisation clinics. Collection of nasal samples takes 5 seconds and causes mild discomfort. The nasal samples will be tested for pneumococcus in Basse and some also tested in London. Samples will be stored for future possible tests relating to infection and may be sent overseas for analyses.

In addition to the main PVS study, your child has been selected for a sub-study to see if the booster dose of pneumococcal vaccine at 9 months of age has a greater effect than no booster dose to prevent children carrying pneumococcal bacteria in the nose. Your child would have 15 nasopharyngeal swabs, at the ages of 6 weeks and 6, 9, 10, 11, 12, 13, 14, 18, 23, 24, 25, 26, 27 and 28 months that we would test in Basse for pneumococcal bacteria. This sub-study will also compare the immune response to the two different pneumococcal vaccine schedules and the immune response to yellow fever vaccine when it is given on the same day as the pneumococcal vaccine or on a separate day. Your child would have pneumococcal vaccine at 9 months of age and yellow fever vaccine at 10 months of age. Your child would have three blood collections of one to two teaspoons. Blood at the ages of 9 and 10 months would be tested for pneumococcal antibodies. Yellow fever antibodies would be tested at 11 months of age. Samples will be sent overseas for testing.

We will provide treatment if there are any problems caused by study procedures. If it is an emergency please go to your nearest health centre. If you have any concerns you can contact Mr Mustapha Darboe on 2225831 or Mr Alhagie Konateh on 7127829.

We will ensure the safety of the study by continuous monitoring for pneumococcal disease. If the study needs to be stopped early, we will tell you, and your village members will be given the more effective schedule.

You will not get paid by the study. You are free to let your child join or not and you are free to stop taking part at any time without giving a reason. If your child doesn’t take part the current schedule will be given along with normal medical care. If you do not want your child to continue in the study we will use only the samples and information already collected from your child. If we find any new information during the study that may change whether your child can be in the study, we will inform you as soon as possible.

All information will be kept confidential. Personal information will only be available to selected members of the study team, Sponsor, Ethics Committee and Government authorities. Unidentified information may be used via public, or other data storage, authorised by MRCG at LSHTM. The results will be presented to your community.

This study has been checked by the GG/MRC Ethics Committee and the LSHTM Ethics Committee. The Ethics Committees protect your rights and wellbeing, and have given permission for the study.

**Consent Form**

Participant’s Name Participant’s ID Sticker

*(place identical ID stickers here*

*__________________________________________ and on child welfare record)*

**OR**

(Printed name of parent)

(Printed name of guardian)

I have read the written information **OR**

I have heard the information explained to me in  Serahule,  Fula,  Mandinka,  Wolof, and I

- confirm that my choice to let my child participate is entirely voluntarily,
- confirm that I have had the opportunity to ask questions about this study and I am happy with the answers that have been provided,
- understand that I allow access to information about my child by the persons described in the information sheet,
- had enough time to think about whether I want my child take part in this study,
- agree to allow my child take part in this study.

*Tick as appropriate*

| I agree for 15 nasopharyngeal swabs to be collected while my child is well.  I agree for three blood samples to be collected while my child is well.  I agree for my child’s samples to be shipped outside The Gambia.  I agree to further research on my child’s samples.  I agree to de-identified data being used via MRCG authorised data storage. | | | | Yes  Yes  Yes  Yes  Yes | | No  No  No  No  No |
| --- | --- | --- | --- | --- | --- | --- |
| Participant’s parent/guardian signature/thumbprint* |  |  |  | |  | |
|  |  |  | Date (dd/mmm/yyyy) Time (24hr) | | | |
| Printed name of impartial witness* |  | | | | | |
| Impartial witness’s* signature/thumbprint |  |  |  | |  | |
|  |  |  | Date (dd/mmm/yyyy) Time (24hr) | | | |
| Printed name of person obtaining consent |  | | | | | |
| **I attest that to the best of my knowledge the study information was understood by the parent/guardian and that he/she has freely given consent to participate *in the presence of the above named impartial witness.** | | | | | | |
| Signature of person obtaining consent |  |  |  | | | |
|  |  |  | Date (dd/mmm/yyyy) Time (24hr) | | | |

**Only required if the participant is unable to read or write.*

A copy of this informed consent document has been provided for the participant.
